# Protective effects of sodium dodecyl sulfate-modified chitosan against subchronic mercury-induced hepatorenal toxicity in male swiss albino mice

**DOI:** 10.1016/j.toxrep.2026.102339

**Published:** 2026-09-08

**Authors:** Sepide Safari, Hossein Nourani, Amir Moghaddam Jafari

**Affiliations:** a Department of Basic Sciences, Faculty of Veterinary Medicine, Ferdowsi University of Mashhad, Mashhad, Iran; b Department of Pathobiology, Faculty of Veterinary Medicine, Ferdowsi University of Mashhad, Mashhad, Iran

**Keywords:** Mercury toxicity, SDS-modified chitosan, Hepatorenal dysfunction, Metal chelation

## Abstract

Mercury (Hg) is a persistent environmental trace element that accumulates in biological tissues and induces severe hepatorenal toxicity primarily through oxidative stress–mediated mechanisms. The present study investigated the protective efficacy of a sodium dodecyl sulfate–modified chitosan derivative (SDSCS) against subchronic Hg-induced toxicity in male Swiss albino mice. Animals were orally exposed to mercuric chloride (HgCl₂, 0.5 mg/kg/day), SDSCS (20 mg/kg/day), or their combination for eight weeks. Mercury accumulation in liver and kidney was quantified using hydride-generation atomic absorption spectrophotometry. Biochemical indices of liver and kidney function, oxidative stress markers, antioxidant capacity, hematological parameters, relative organ weights, and histopathological injury scores were evaluated. Hg exposure resulted in marked accumulation of mercury in the kidney and liver, accompanied by significant increases in lipid peroxidation, liver enzymes (ALT, AST), renal dysfunction markers (BUN, creatinine), relative organ weights, and histopathological damage, alongside depletion of total antioxidant capacity (FRAP). SDSCS co-administration significantly reduced tissue mercury burden, attenuated oxidative stress, partially restored biochemical and hematological alterations, and improved histological architecture. Multivariate Z-score and Pearson correlation analyses revealed strong positive associations between mercury accumulation, oxidative stress, organ hypertrophy, functional impairment, and tissue injury, while antioxidant capacity exhibited inverse relationships with toxicity indices. Collectively, these findings indicate that SDSCS mitigates Hg-induced hepatorenal toxicity by limiting tissue mercury accumulation and supporting redox balance, consistent with its potential as a biopolymer-based intervention against mercury toxicity.

## Highlights

• SDS-modified chitosan significantly reduced mercury-induced hepatorenal toxicity in mice.
• SDSCS decreased hepatic and renal mercury accumulation through effective metal chelation.
• Mercury exposure induced severe oxidative stress and antioxidant depletion in liver and kidney tissues.
• SDSCS restored antioxidant defenses and improved biochemical and histopathological alterations.
• Integrated multivariate analyses confirmed coordinated protection against systemic mercury toxicity.

## Graphical Abstract

**Figure:**
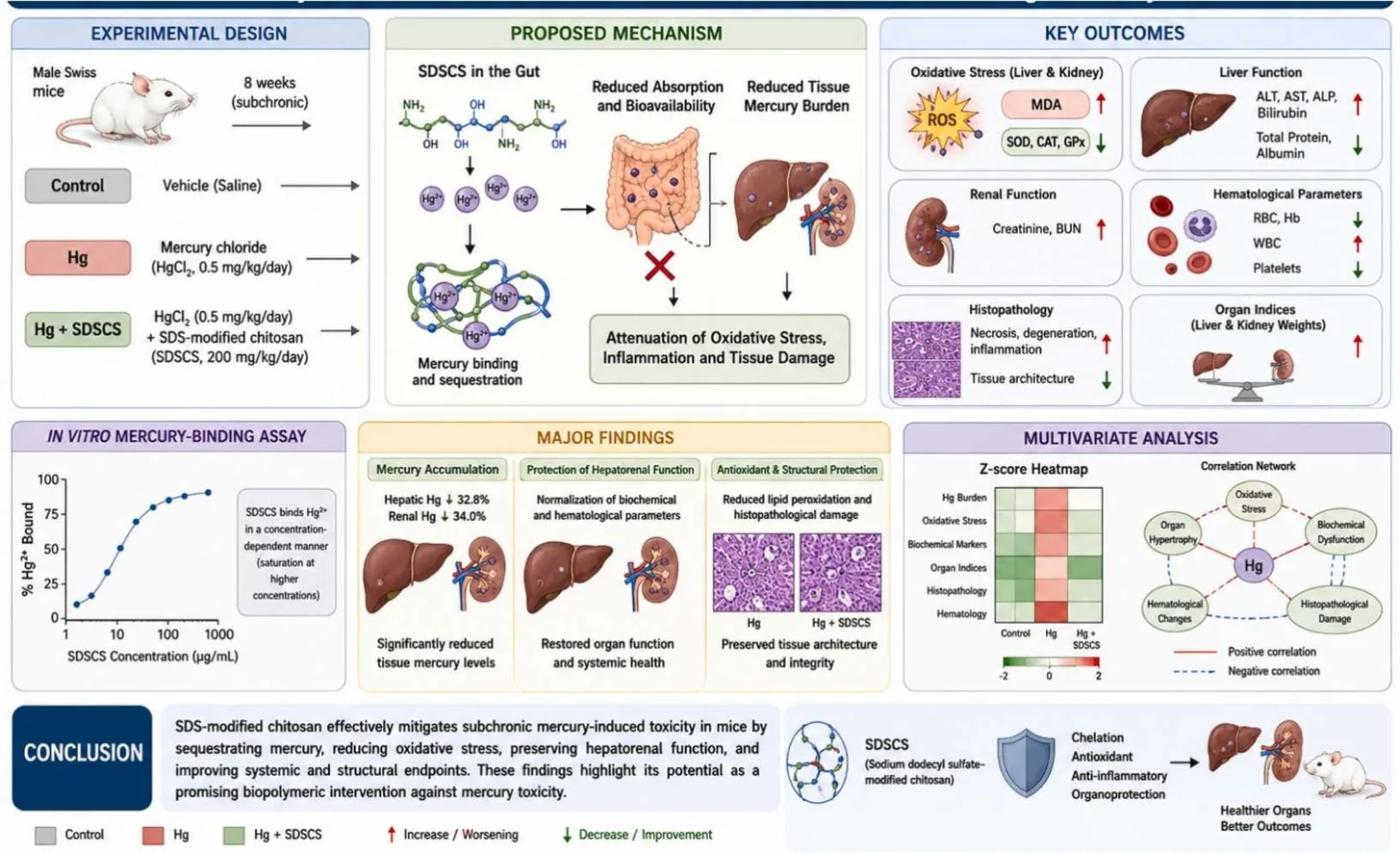

## 1 Introduction

Mercury poisoning remains a significant environmental and public health concern because of its persistence, global distribution, and high toxicity. Mercury is released from natural processes and anthropogenic activities, including artisanal gold mining, coal combustion, chlor-alkali industries, waste incineration, and industrial emissions, resulting in continuous contamination of aquatic and terrestrial ecosystems [2], [8], [45]. Human exposure occurs through occupational contact, contaminated food—particularly fish and seafood—and environmental pollution [6], [57]. Mercury exists as elemental, inorganic, and organic species, each possessing distinct toxicokinetic characteristics but sharing a remarkable affinity for sulfhydryl (-SH) groups of proteins. This interaction disrupts numerous cellular processes and promotes accumulation within metabolically active organs, particularly the liver, kidneys, and central nervous system [58]. Mercury-induced toxicity is now recognized as a multifactorial process involving oxidative stress, mitochondrial dysfunction, inflammatory activation, disruption of calcium homeostasis, apoptosis, and impairment of cellular energy metabolism. Among these mechanisms, oxidative stress represents the central event linking mercury exposure to downstream tissue injury. Mercury depletes intracellular glutathione, inhibits endogenous antioxidant enzymes including superoxide dismutase, catalase, and glutathione peroxidase, and promotes excessive reactive oxygen species (ROS) generation, ultimately leading to lipid peroxidation, protein oxidation, DNA damage, mitochondrial dysfunction, and cell death [7], [22].

The liver serves as the principal organ for xenobiotic metabolism, whereas the kidneys represent the primary site of inorganic mercury accumulation and excretion. Consequently, these organs are particularly vulnerable to mercury-induced injury, which manifests as hepatocellular degeneration, tubular necrosis, impaired liver and kidney function, and progressive histopathological damage [20], [32]. Chronic mercury exposure has also been associated with neurotoxicity, cardiovascular dysfunction, reproductive impairment, and immune dysregulation, highlighting its broad toxicological significance [3], [4].

Although oxidative stress is considered the principal mechanism of mercury toxicity, similar redox-dependent pathways also contribute to tissue injury induced by other persistent heavy metals, including cadmium, lead, and hexavalent chromium. Comparative toxicological studies indicate that oxidative stress, inflammation, and mitochondrial dysfunction constitute common pathogenic mechanisms across heavy metal exposures, although each metal exhibits distinct toxicokinetic behavior and organ specificity. These observations emphasize the importance of developing broad-spectrum strategies capable of reducing heavy metal bioavailability while simultaneously attenuating oxidative damage.

Current clinical management of mercury poisoning relies primarily on chelating agents such as dimercaptosuccinic acid (DMSA), 2,3-dimercapto-1-propanesulfonic acid (DMPS), and British anti-Lewisite (BAL). Despite their established efficacy, these therapies possess important limitations, including limited tissue penetration, incomplete removal of intracellular mercury, redistribution of mercury among tissues, repeated dosing requirements, and potential adverse effects. Consequently, there is increasing interest in safer biomaterial-based approaches that may reduce mercury absorption and bioaccumulation while exhibiting superior biocompatibility.

Among natural biopolymers, chitosan has attracted considerable attention because of its biodegradability, low toxicity, excellent biocompatibility, and abundant amino and hydroxyl functional groups capable of coordinating metal ions [33], [44]. Previous investigations have demonstrated that native chitosan and several chemically modified derivatives effectively adsorb and chelate heavy metals including cadmium, lead, chromium, and mercury [10], [33]. Chemical modification of chitosan with sodium dodecyl sulfate (SDS) substantially alters its physicochemical properties by increasing surface activity, improving aqueous dispersion, exposing additional functional groups, and enhancing interactions between mercury ions and the amino/hydroxyl binding sites of the polymer matrix. These modifications may improve metal-binding efficiency and biological performance compared with native chitosan. However, despite extensive environmental applications of modified chitosan materials, relatively few in vivo studies have investigated their protective efficacy against mercury-induced systemic toxicity.

Therefore, the present study was designed to comprehensively evaluate the protective effects of SDS-modified chitosan (SDSCS) against subchronic inorganic mercury toxicity in male Swiss albino mice. The study integrated in vitro mercury-binding assays with in vivo determination of tissue mercury accumulation, biochemical and hematological analyses, oxidative stress assessment, antioxidant capacity, organ morphometry, and histopathological evaluation. Furthermore, integrated Z-score profiling and Pearson correlation heatmap analyses were employed to characterize the relationships among mercury burden, oxidative injury, organ dysfunction, and tissue pathology, thereby providing a systems-level evaluation of mercury toxicity.

The novelty of this work lies in the integration of concentration-dependent in vitro mercury chelation, quantitative tissue mercury determination, comprehensive toxicological assessment, and multivariate statistical analyses within a single experimental framework. Rather than establishing a definitive therapeutic mechanism, the present findings provide strong preclinical evidence supporting SDS-modified chitosan as a promising biomaterial for further investigation as a candidate strategy to reduce mercury bioaccumulation and mitigate mercury-induced hepatorenal toxicity.

## 2 Materials and methods

### 2.1 Chemicals

Mercuric chloride (HgCl₂), 2-thiobarbituric acid (TBA), sodium hydroxide (NaOH), sodium chloride (NaCl), sodium acetate trihydrate (C₂H₃NaO₂·3H₂O), glacial acetic acid, ferric chloride hexahydrate (FeCl₃·6H₂O), ferrous sulfate heptahydrate (FeSO₄·7H₂O), 2,4,6-tris(2-pyridyl)-s-triazine (TPTZ), hydrochloric acid (HCl), and potassium chloride (KCl) were purchased from Merck (Darmstadt, Germany). All chemicals were of analytical grade and used without further purification.

### 2.2 Experimental animals

Forty healthy adult male Swiss albino mice (8–10 weeks old; body weight 20–25 g) were obtained from the Animal House of the Avicenna Research Institute (Mashhad, Iran). Only male mice were included to minimize variability associated with hormonal fluctuations during the estrous cycle, thereby improving statistical consistency in oxidative stress, biochemical, and histopathological endpoints. Animals were housed in polypropylene cages (7 animals/cage) under controlled environmental conditions (temperature 25 ± 1°C, relative humidity 50 ± 5%, 12-h light/12-h dark cycle). Before initiation of the experiment, mice were acclimatized for one week with free access to standard laboratory chow and drinking water. Animals had ad libitum access to food and water throughout the 8-week study. Animals were randomly allocated into experimental groups using a computer-generated randomization schedule. Daily health status, food consumption, and clinical signs of toxicity were monitored throughout the study. At the end of the experimental period, mice were euthanized by gradual-fill carbon dioxide (CO₂) inhalation in a euthanasia chamber. Death was confirmed by the absence of respiration and heartbeat before tissue collection. All procedures complied with the Guide for the Care and Use of Laboratory Animals and were approved by the Animal Welfare Committee of the Faculty of Veterinary Medicine, Ferdowsi University of Mashhad (Ethical approval No. IR.UM.REC.1400.102).

### 2.3 Preparation of SDS-modified chitosan (SDSCS)

The preparation of sodium dodecyl sulfate-modified chitosan (SDSCS) was performed according to our previously published protocol with slight modifications [33]. Chitosan powder (16 g) was dissolved in 800 mL of 1% (v/v) acetic acid under continuous magnetic stirring. Subsequently, 0.5 M sodium hydroxide solution was added slowly until gel formation occurred. The resulting gel was stirred at 300 rpm for 20 min, followed by addition of 0.3% (w/v) SDS solution. The suspension was stirred for an additional 60 min at room temperature (25 ± 2°C). The precipitated polymer was filtered, washed repeatedly with distilled water until neutral pH, dried overnight at 60°C, ground, and sieved. Based on elemental analysis, the final material contained approximately 2% (w/w) SDS. Physicochemical characterization was performed using Fourier-transform infrared spectroscopy (FTIR) and scanning electron microscopy (SEM), confirming successful modification of chitosan by SDS and alterations in surface morphology compared with native chitosan [33].

### 2.4 In vitro mercury-chelation assay

The mercury-binding capacity of SDSCS was evaluated using a concentration-dependent in vitro assay. Mercuric chloride solution (10 mg/L Hg²⁺) was freshly prepared using deionized water. SDSCS was added at final concentrations of 0.5, 1.0, 1.5, and 2.0% (w/v). Each reaction mixture (20 mL) was adjusted to pH 7.0 ± 0.1 and incubated at room temperature (25 ± 2°C) under continuous magnetic stirring (300 rpm) for 60 min to ensure equilibrium between mercury ions and the polymer. All experiments were performed in triplicate (n = 3 independent experiments)**.** Following incubation, suspensions were centrifuged at 5000 × g for 10 min, and the supernatants were collected. Residual mercury concentration was quantified using hydride-generation atomic absorption spectrophotometry (HG-AAS, GBC Scientific Equipment, Australia). Mercury-binding efficiency was calculated using the following equation:

Hg binding (%) = (C_0_ – C_f_)/ C_0_ × 100

where C₀ represents the initial mercury concentration and C_f_ the residual mercury concentration after incubation. Statistical comparisons among SDSCS concentrations were performed using one-way ANOVA followed by Tukey's multiple-comparison test. Saturation of mercury binding was assessed by evaluating changes in binding efficiency across increasing SDSCS concentrations.

### 2.5 Experimental design

Following acclimatization, mice were randomly assigned (n = 10/group) into four experimental groups:

1. **Control:** distilled water (0.5 mL/day)
2. **SDSCS:** SDSCS (20 mg/kg/day)
3. **Hg:** HgCl₂ (0.5 mg/kg/day)
4. **Hg + SDSCS:** HgCl₂ (0.5 mg/kg/day) plus SDSCS (20 mg/kg/day)

All treatments were administered once daily by oral gavage for eight consecutive weeks. Oral gavage was selected to ensure accurate dose delivery throughout the study. The selected mercury dose (0.5 mg/kg/day) was based on established subchronic rodent models producing reproducible hepatorenal injury without excessive mortality and corresponds to a human equivalent dose of approximately 0.04 mg/kg/day using body surface area conversion. The SDSCS dose (20 mg/kg/day) was selected based on our previous toxicity and physicochemical characterization study demonstrating its excellent biocompatibility and absence of detectable systemic toxicity [34], together with preliminary pilot experiments showing effective mercury-binding capacity without adverse effects. Investigators responsible for biochemical measurements and histopathological scoring were blinded to treatment allocation to minimize observational bias.

### 2.6 Measurement of body and organ weight

Body weight was recorded weekly throughout the 8-week experimental period using a calibrated electronic balance (AND GX-600, Japan). Animals were observed daily for general health status, food intake, water consumption, and clinical signs of toxicity. At the end of the treatment period, mice were euthanized by CO₂ inhalation followed by cervical dislocation to ensure death, in accordance with institutional ethical guidelines. The liver, kidneys, brain, spleen, and heart were immediately excised, carefully rinsed with ice-cold physiological saline to remove residual blood, gently blotted dry with filter paper, and weighed individually using an analytical balance. Both absolute organ weights (g) and relative organ weights (%) were calculated. Relative organ weight was determined using the following equation:

Relative Organ Weight (%) = {Absolute Organ Weight (g) / Body Weight (g)}× 100

Relative organ weight was used as an indicator of organ hypertrophy or atrophy associated with mercury-induced toxicity.

### 2.7 Evaluation of hematological and biochemical parameters

Whole blood was collected immediately after euthanasia by cardiac puncture under deep anesthesia. Blood samples were divided into two portions. One portion was collected into EDTA-containing tubes for hematological analysis, whereas the second portion was allowed to clot at room temperature for serum preparation. After clot formation, samples were centrifuged at 3000 × g for 10 min at 4°C, and the obtained serum was stored at −20°C until biochemical analysis**.** Hematological parameters, including red blood cell (RBC) count, hemoglobin (Hb), hematocrit (Hct), mean corpuscular hemoglobin (MCH), mean corpuscular volume (MCV), mean corpuscular hemoglobin concentration (MCHC), platelet count, total white blood cell (WBC) count, and differential leukocyte count, were measured using an automated hematology analyzer (Nihon Kohden Celtac Alpha, Japan). Serum biochemical analyses, including glucose, lipid profile (total cholesterol, triglycerides, HDL-C, LDL-C and VLDL-C), liver function biomarkers (ALT, AST, ALP, total bilirubin, total protein, albumin and globulin), and renal function biomarkers (blood urea nitrogen and creatinine), were determined using commercial diagnostic kits (Pars Azmoon Co., Tehran, Iran) on an automated chemistry analyzer (Targa 3000, Italy), following the manufacturer's instructions. All biochemical analyses were performed in duplicate, and routine internal quality control sera were analyzed before each analytical run to ensure assay accuracy and precision.

### 2.8 issue oxidative stress marker assays

#### 2.8.1 Tissue preparation

Immediately after organ collection, liver and kidney tissues were rinsed thoroughly with ice-cold phosphate-buffered saline (PBS) to remove residual blood and connective tissue. Approximately 100 mg of each tissue was weighed and homogenized in 10 volumes (10%, w/v) of ice-cold 1.15% KCl prepared in 0.01 M sodium phosphate buffer (pH 7.4) using a Silent Crusher M homogenizer (Heidolph Instruments GmbH & Co. KG, Germany). The homogenates were centrifuged at 18,000 × g for 20 min at 4°C, and the resulting supernatants were collected and immediately used for oxidative stress assays or stored at −80°C until analysis**.** All procedures were performed on ice to minimize artificial oxidation during sample preparation.

#### 2.8.2 Lipid peroxidation assay (TBARS)

Lipid peroxidation was evaluated by measuring malondialdehyde (MDA) concentrations using the thiobarbituric acid reactive substances (TBARS) assay. Briefly, 0.2 mL of tissue homogenate was mixed with 1.3 mL of Tris–KCl buffer (0.2 M Tris-HCl containing 0.16 M KCl, pH 7.4) and 1.5 mL of thiobarbituric acid reagent. The reaction mixture was heated in a boiling water bath **(**100°C**)** for 10 min, cooled to room temperature, and extracted with 3 mL pyridine:n-butanol (3:1, v/v) followed by 1 mL of 1 N NaOH. After vigorous mixing and phase separation, absorbance was measured at 548 nm using a UV–visible spectrophotometer against an appropriate reagent blank. MDA concentrations were calculated using an extinction coefficient of 1.56 × 10⁵ M⁻¹ cm⁻¹ and expressed as nmol MDA/mg tissue**.** All samples were analyzed in triplicate, and intra-assay variation was < 5%.

#### 2.8.3 Total antioxidant capacity (FRAP assay)

Total antioxidant capacity (TAC) was determined using the ferric reducing antioxidant power (FRAP) assay as originally described by Benzie and Strain with minor modifications. The assay is based on the reduction of the ferric-tripyridyltriazine (Fe³⁺–TPTZ) complex to the ferrous form (Fe²⁺–TPTZ) under acidic conditions, producing an intense blue color. The freshly prepared FRAP reagent consisted of acetate buffer (300 mM, pH 3.6), TPTZ solution (10 mM dissolved in 40 mM HCl), and FeCl₃·6H₂O (20 mM) mixed at a ratio of 10:1:1. After incubation for 5 min at 37°C, absorbance was measured at 593 nm. A calibration curve was prepared using freshly prepared FeSO₄·7H₂O standards (100–1000 μM), and results were expressed as μmol Fe²⁺ equivalents per gram of tissue.Each sample was analyzed in triplicate, and all measurements were performed within the linear range of the standard curve.

### 2.9 Histopathological examination

Liver and kidney tissues were immediately excised after euthanasia and fixed in 10% neutral-buffered formalin for 24–48 h. The tissues were subsequently dehydrated through a graded ethanol series, cleared in xylene, embedded in paraffin wax, and sectioned at 5–6 μm thickness using a rotary microtome. Tissue sections were stained with hematoxylin and eosin (H&E) following standard histological procedures. Histological examination was performed using a light microscope (Labomed LX400, USA) equipped with a digital camera (TrueChrome II, Labo America Inc., USA). All histopathological evaluations were performed by a veterinary pathologist who was blinded to the treatment groups to minimize observer bias. Histopathological alterations, including hepatocellular or tubular degeneration (cell swelling, cytoplasmic vacuolation, and ballooning), necrosis (loss of cellular architecture, karyolysis, pyknosis, and karyorrhexis), vascular congestion, and inflammatory cell infiltration, were evaluated using a semi-quantitative scoring system:

• 0 = no detectable lesion
• 1 = lesions involving < 25% of the examined field
• 2 = lesions involving 25–50%
• 3 = lesions involving 50–75%
• 4 = lesions involving > 75%

Six randomly selected non-overlapping microscopic fields per section were evaluated at × 200 magnification, and the total histopathological score was calculated as the sum of individual lesion scores.

### 2.10 Determination of mercury concentration in liver and kidney tissues

#### 2.10.1 Sample preparation

Approximately 1.0 g of liver or kidney tissue was collected from each animal. Samples were dried at 135°C for 2 h to constant weight. Approximately 0.5 g of dried tissue was accurately weighed and transferred into acid-washed glass digestion vessels. Each sample received 10 mL of concentrated nitric acid (65%, analytical grade) and was allowed to undergo overnight predigestion at room temperature. Samples were then heated gently until complete digestion was achieved and a clear solution was obtained. The digested samples were filtered through 0.45 μm membrane filters and diluted to a final volume of 25 mL using 0.1 N nitric acid. Mercury concentrations were subsequently determined by hydride-generation atomic absorption spectrophotometry (HG-AAS) and expressed as ppb per dry tissue weight.

#### 2.10.2 Atomic absorption spectrophotometric analysis

Mercury concentrations were determined using a hydride generation system coupled to a flame atomic absorption spectrophotometer (HG-AAS). The hydride generation unit was mounted on the flame atomizer, and a dedicated quartz absorption cell was installed in the optical path for mercury vapor detection. Sodium borohydride was used as the reducing agent to generate elemental mercury vapor from the digested samples. Before analysis, the quartz cell alignment was optimized to maximize detector response. Instrument calibration was performed using freshly prepared mercury standard solutions (15, 20, and 30 ppb), each analyzed in triplicate. Calibration curves exhibited excellent linearity throughout the working range (R² = 0.999). Following calibration, quality control (QC) samples were analyzed before and periodically during sample analysis to verify analytical accuracy and instrument stability. Mercury concentrations in unknown samples were calculated from the calibration curve after correction for dilution factors and sample dry weight. The analytical limit of detection (LOD) and limit of quantification (LOQ) were determined as 0.5–2ppb and 1–5 ppb, respectively. Method accuracy was assessed by recovery analysis using mercury-spiked tissue samples, yielding a mean recovery of 90–110%. Analytical precision was evaluated by triplicate measurements.

#### 2.10.3 Calibration curve and quantification

Standard mercury solutions were prepared, and a calibration curve (Table-1) was generated using three different standard concentrations, each measured in triplicate. Absorbance readings were recorded, and mercury concentrations in unknown samples were calculated based on the calibration curve, taking into account dilution factors and the initial sample weight.

**Table 1:** Calibration (standard) curve data for mercury determination by hydride generation atomic absorption spectrophotometry (HG-AAS).

**Standard No.**	**Mercury Concentration (ppb)**	**Absorbance**
1	15	0.215
2	20	0.334
3	30	0.537

### 2.11 Statistical analysis

Statistical analyses were performed using SPSS version 11.5 (SPSS Inc., Chicago, IL, USA). Prior to statistical analysis, data normality was assessed using the Shapiro–Wilk test, and homogeneity of variances was evaluated using Levene's test. Normally distributed data were analyzed by one-way analysis of variance (ANOVA) followed by Tukey's multiple comparison test. Continuous data are presented as mean ± standard error of the mean (SEM). Histopathological scores were analyzed using the Kruskal–Wallis test, followed by the Mann–Whitney *U* test for pairwise comparisons because these data were not normally distributed. Histopathological results are presented as median (minimum–maximum). Differences were considered statistically significant at p < 0.05.

### 2.12 Integrated Z-score and correlation heatmap analysis

To visualize the overall pattern of mercury-induced toxicity and the protective effects of SDSCS, multivariate heatmap analyses were performed. Variables included in the analysis were selected a priori based on their biological relevance and included tissue mercury concentrations, relative liver and kidney weights, oxidative stress biomarkers (MDA and FRAP), biochemical markers of liver and kidney function, and histopathological injury scores. Prior to analysis, all variables were standardized using Z-score normalization to eliminate differences in measurement scales. Pearson correlation coefficients were subsequently calculated to evaluate associations among variables. Only statistically significant correlations (p < 0.05) were displayed in the correlation heatmap.

## 3 Results

### 3.1 In vitro mercury-binding capacity of SDSCS

The in vitro mercury-binding assay demonstrated a significant concentration-dependent increase in the mercury-chelating capacity of SDSCS (Table 2). At the lowest concentration (0.5%, w/v), SDSCS removed approximately 21.5% of mercury from solution, whereas increasing the concentration to 1.0% resulted in 70.3% mercury removal. Further increases to 1.5% and 2.0% (w/v) enhanced the binding efficiency to 85.0% and 95.5%, respectively.

**Table 2:** Effect of increasing SDSCS concentrations on In vitro mercury binding. Mercury-binding capacity was evaluated at different concentrations of sodium dodecyl sulfate-modified chitosan (SDSCS; 0–2% w/v) using a fixed initial mercury concentration. Residual mercury represents the mercury concentration remaining in the reaction solution after treatment, whereas Hg binding (%) represents the percentage of mercury removed from the solution by SDSCS. Data are presented as mean ± SEM. Different superscript letters within the same column indicate statistically significant differences among SDSCS concentrations according to one-way ANOVA followed by Tukey–Kramer post hoc test (p < 0.05).

**SDSCS concentration (% w/v)**	**Residual Hg (mg/L)**	**Hg binding (%)**
0 (Hg only)	10.00 ± 0.18^a^	0^a^
0.5%	7.90 ± 0.22^b^	21.5 ± 2.1^b^
1%	3.02 ± 0.19^c^	70.3 ± 1.9^c^
1.5%	2.50 ± 0.17^d^	85 ± 2.4^d^
2%	0.5 ± 0.05^e^	95.5 ± 2.8^e^

The progressive increase in mercury removal reached an apparent plateau at the highest concentration tested, suggesting saturation of the available mercury ions rather than polymer binding sites under the experimental conditions. Statistical analysis confirmed significant differences among SDSCS concentrations (p < 0.05). These findings demonstrate the strong mercury-binding capacity of SDSCS and support its subsequent evaluation in the in vivo model.

### 3.2 Effects of Hg and SDSCS on body weight and organ indices

**Fig. 1:**
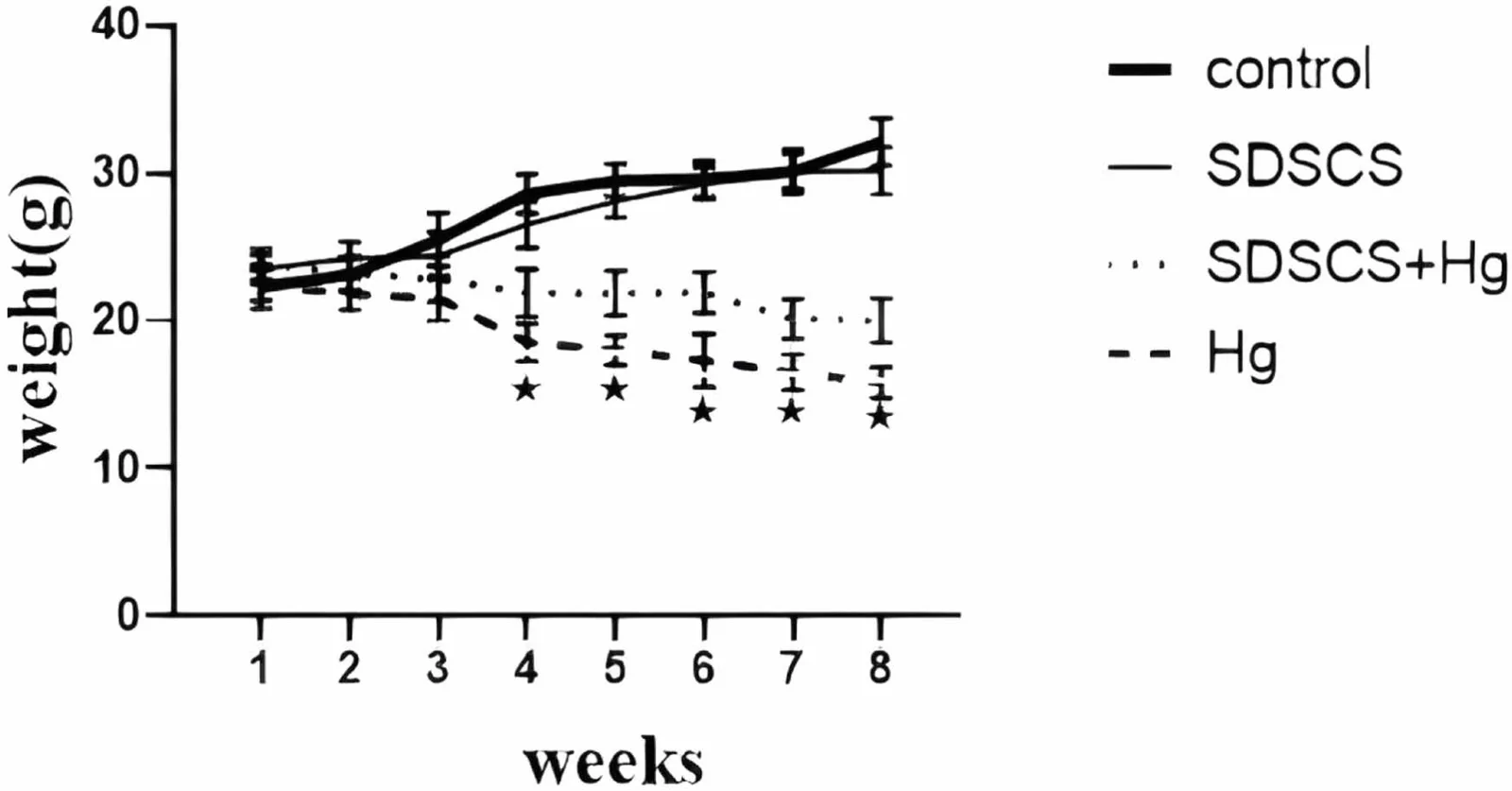
Effects of Hg (0.5 mg/kg/day), SDSCS (20 mg/kg/day), and combined treatment on body weight changes in male Swiss albino mice during the 8-week experimental period. Compounds were administered once daily by oral gavage. Values are presented as mean ± SEM (n = 10 per group). Statistical analysis was performed using one-way ANOVA followed by Tukey's multiple comparison test. ^a^ p < 0.05 vs. Control; ^b^ p < 0.05 vs. Hg group.

**Fig. 2:**
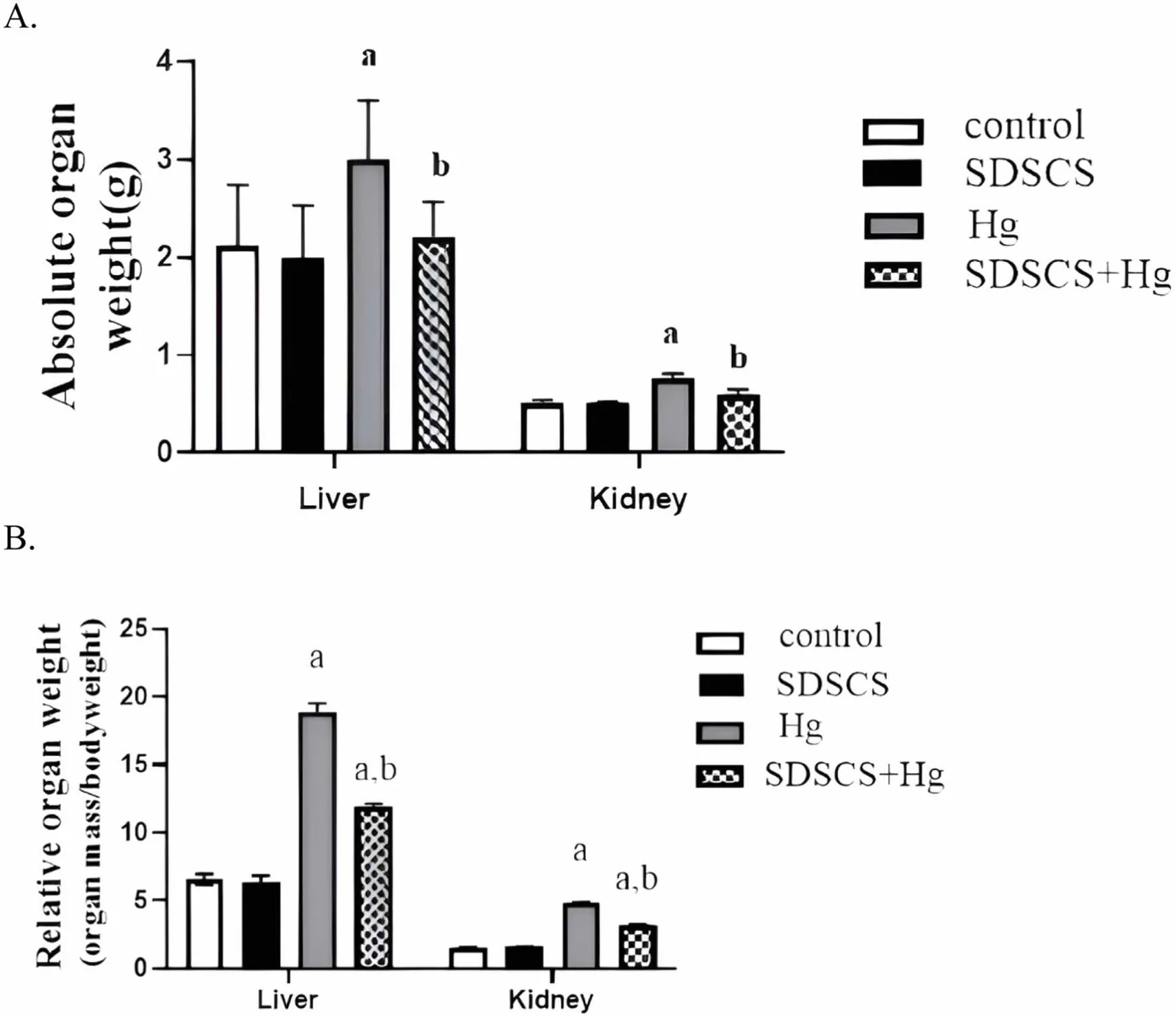
Effects of Hg (0.5 mg/kg/day), SDSCS (20 mg/kg/day), and combined treatment on absolute (A) and relative (B) liver and kidney weights in male Swiss albino mice following 8 weeks of oral exposure. Values are expressed as mean ± SEM (n = 10 per group). Statistical analysis was performed using one-way ANOVA followed by Tukey's multiple comparison test. ^a^ p < 0.05 vs. Control; ^b^ p < 0.05 vs. Hg group.

No mortality or overt clinical signs of toxicity were observed in any experimental group during the 8-week exposure period. Mercury exposure significantly reduced body weight gain beginning in week 4, with progressively greater reductions throughout the study (p < 0.05, Fig. 1). Body weight remained significantly lower than controls through week 8. In contrast, mice receiving SDSCS alone did not differ from the control group, whereas co-administration of SDSCS significantly attenuated Hg-induced body weight loss, shifting body weight toward control values (p < 0.05 vs. Hg group). Mercury exposure also produced marked increases in both absolute and relative liver and kidney weights **(**Fig. 2**).** Compared with controls, relative liver and kidney weights increased by approximately 188% and 204%, respectively (p < 0.05). Animals receiving SDSCS together with mercury exhibited significantly lower organ weights than the Hg-treated group **(**p < 0.05**),** although values remained modestly higher than those of controls. No significant differences were observed between the SDSCS-only and control groups. These findings indicate that SDSCS significantly alleviated Hg-induced alterations in body weight and organ hypertrophy.

### 3.3 Impact of Hg and SDSCS Treatment on Hematological Parameters

Subchronic mercury exposure produced marked hematological alterations in male Swiss albino mice (Table 3). Compared with the control group, Hg significantly increased total leukocyte count, primarily because of elevations in neutrophils, lymphocytes, and monocytes (p < 0.05), indicating a systemic inflammatory response. Co-administration of SDSCS significantly reduced leukocyte counts relative to the Hg group, shifting these parameters toward control values, although complete normalization was not observed for all leukocyte subsets. Mercury exposure also induced hematological evidence of anemia and thrombocytopenia, characterized by significant reductions in erythrocyte count, hemoglobin concentration, hematocrit, and platelet count, together with increased mean corpuscular volume (MCV) and decreased mean corpuscular hemoglobin concentration (MCHC) (p < 0.05**).** SDSCS treatment significantly improved these erythrocyte-related parameters compared with Hg exposure alone, indicating partial restoration of erythropoiesis and platelet homeostasis. No significant differences were observed between the SDSCS-alone and control groups for any hematological parameter (p > 0.05), demonstrating that SDSCS itself did not adversely affect hematological homeostasis.

**Table 3:** Effect of HgCl₂ (0.5 mg/kg/day), SDSCS (20 mg/kg/day), and combined treatment on hematological parameters in male Swiss albino mice after 8 weeks of oral gavage. Data are presented as mean ± SEM (n = 10 per group). Statistical comparisons were performed using one-way ANOVA followed by Tukey's post hoc test. ^a^ p < 0.05 versus Control; ^b^ p < 0.05 versus Hg group.

Parameters	Control	SDSCS	Hg	SDSCS + Hg
WBC (*10^**3**^**/uL)**	6.34 ± 0.92	6.02 ± 0.82	11.5 ± 0.73^a^	8.7 ± 0.88^a,b^
Neu (*10^**3**^**/uL)**	1.20 ± 0.58	1.15 ± 0.66	2.10 ± 0.50^a^	1.79 ± 0.54^a,b^
Lym (*10^**3**^**/uL)**	4.88 ± 0.87	4.45 ± 0.92	8.36 ± 0.44^a^	6.52 ± 0.81^a,b^
Mono (*10^**3**^**/uL)**	0.15 ± 0.03	0.13 ± 0.02	0.29 ± 0.06^a^	0.13 ± 0.04
RBC (*10^**6**^**/uL)**	8.2 ± 0.42	7.98 ± 0.53	5.73 ± 0.82^a^	7.68 ± 0.61
**Hemoglobin (g/dL)**	13.2 ± 1.02	12.86 ± 1.16	8.26 ± 1.15^a^	11.96 ± 1.72
**Hematocrit(%)**	40.2 ± 1.5	40.3 ± 1.6	32.81 ± 1.7^a^	39. 5 ± 1.6
**MCH (pg)**	15.3 ± 0.35	15.1 ± 0.55	15.0 ± 0.63	15.0 ± 0.33
**MCV (fL)**	49.2 ± 3.49	50.3 ± 2.50	68.4 ± 3.72^a^	48.5 ± 2.86
**MCHC (g/dL)**	32.2 ± 1.56	33.8 ± 0.92	27.3 ± 0.96^a^	31.7 ± 0.91
Thrombocyte(*10/µL)	1.16 ± 0.62	1.08 ± 0.92	0.68 ± 0.43^a^	1.00 ± 0.81

### 3.4 Impact of Hg and SDSCS treatment on hepatic function

Oral exposure to HgCl₂ for 8 weeks significantly impaired hepatic function, as demonstrated by marked increases in serum ALT, AST, ALP, and total bilirubin levels, together with significant reductions in total protein and albumin concentrations compared with the control group (p < 0.05; Table 4). Globulin concentrations showed no statistically significant difference among the experimental groups.Co-administration of SDSCS significantly improved all altered hepatic biochemical parameters compared with the Hg-treated group (p < 0.05). Serum transaminases, ALP, and total bilirubin were significantly reduced, whereas total protein and albumin concentrations were partially restored toward control values, although several parameters remained significantly different from controls. No significant differences were observed between the SDSCS-only and control groups (p > 0.05). Overall, these findings demonstrate that SDSCS markedly attenuated Hg-induced hepatic dysfunction, although complete restoration of liver function was not achieved under the present experimental conditions (Table 4).

**Table 4:** Effect of HgCl₂ and SDSCS on liver function parameters in male Swiss albino mice. Values are expressed as mean ± SEM (n = 10)**.** HgCl₂ (0.5 mg/kg/day) and SDSCS (20 mg/kg/day) were administered by oral gavage for 8 weeks. ^*a*^ p < 0.05 versus Control group, ^b^ p < 0.05 versus Hg group.

Parameters	Control	SDSCS	Hg	SDSCS + Hg
**Total protein (mg/dL)**	6.42 ± 0.31	6.5 ± 0.56	4.2 ± 0.61^a^	5.5 ± 0.23^a,b^
**Albumin (mg/dL)**	2.43 ± 0.13	2.35 ± 0.22	1.03 ± 0.34^a,b^	1.80 ± 0.52^a,b^
**Globulin (mg/dL)**	3.97 ± 0.19	3.78 ± 0.80	3.20 ± 0.40	3.88 ± 0.16
**Total Bilirubin (mg/dL)**	0.60 ± 0.01	0.59 ± 0.03	0.88 ± 0.06^a^	0.58 ± 0.08
**AST (U/L)**	186 ± 3.8	183 ± 4.2	280 ± 8.9^a^	220 ± 4.4^a,b^
**ALT (U/L)** **ALP(U/L)**	46.5 ± 0.5 72.86 ± 86	43.2 ± 1.0 70.96 ± 2.2	139.6 ± 3.6^a^ 115 ± 3.8^a^	89.6 ± 4.8^a,b^ 91.6 ± 1.5^a,b^

### 3.5 Impact of Hg and SDSCS treatment on renal function

Oral exposure to HgCl₂ for 8 weeks significantly impaired renal function, as evidenced by marked increases in serum creatinine and blood urea nitrogen (BUN) concentrations compared with the control group (p < 0.05; Table 5). Co-administration of SDSCS significantly reduced both serum creatinine and BUN levels compared with the Hg-treated group (p < 0.05), indicating partial restoration of renal function. However, these parameters remained significantly different from those of the control group. No significant differences were observed between the SDSCS-only and control groups (p > 0.05). Overall, these findings demonstrate that SDSCS markedly attenuated Hg-induced renal dysfunction, although complete recovery was not achieved under the present experimental conditions (Table 5).

**Table 5:** Effect of HgCl₂ and SDSCS on renal function parameters in male Swiss albino mice. Values are expressed as mean ± SEM (n = 10). HgCl₂ (0.5 mg/kg/day) and SDSCS (20 g/kg/day) were administered by oral gavage for 8 weeks. ^a^ p < 0.05 versus Control group, ^b^ p < 0.05 versus Hg group.

Parameters	Control	SDSCS	Hg	SDSCS + Hg
**BUN (mg/dL)**	25.86 ± 0.62	24.43 ± 0.53	45.20 ± 0.82^a^	33 ± 0.68^a,b^
**Creatinine (m/dL)**	0.55 ± 0.03	0.52 ± 0.03	1.20 ± 0.06^a^	0.82 ± 0.08

### 3.6 Impact of Hg and SDSCS treatment on blood glucose and lipid profile

As shown in Table 6**,** Hg exposure significantly disturbed glucose and lipid metabolism, resulting in elevated serum glucose, total cholesterol, triglycerides, LDL-c, and VLDL-c levels, together with a significant reduction in HDL-c compared with the control group (p < 0.05). Co-treatment with SDSCS significantly improved these metabolic alterations compared with the Hg-treated group (p < 0.05). Blood glucose, total cholesterol, triglycerides, LDL-c, and VLDL-c levels were significantly reduced, whereas HDL-c concentrations were partially restored toward control values**.** Nevertheless, several parameters remained significantly different from those of the control group. The SDSCS-only group did not differ significantly from the control group in any measured metabolic parameter (p > 0.05). Overall, these results indicate that SDSCS effectively attenuated Hg-induced disturbances in glucose homeostasis and lipid metabolism, although complete normalization was not observed (Table 6).

**Table 6:** Effect of HgCl₂ and SDSCS on blood glucose and lipid profile in male Swiss albino mice. Values are expressed as mean ± SEM (n = 10). HgCl₂ (0.5 mg/kg/day) and SDSCS (20 mg/kg/day) were administered by oral gavage for 8 weeks. ^a^ p < 0.05 versus Control group, ^b^ p < 0.05 versus Hg group.

parameters	Control	SDSCS	Hg	SDSCS + Hg
**Glucose (mg/dL)**	86 ± 3.50	88 ± 4.00	138 ± 4.20^a^	100 ± 2.53^a,b^
**Cholesterol (mg/dL)**	68 ± 2.62	69 ± 3.50	108 ± 4.22^a^	89 ± 3.28^a,b^
**Triglyceride (mg/dL)**	134 ± 1.83	132 ± 2.50	189 ± 4.35^a^	160 ± 3.92^a,b^
**HDL-c (mg/dL)**	50.25 ± 0.80	53.30 ± 0.76	30.50 ± 0.83^a^	44.20 ± 0.91^a,b^
**LDL-c (mg/dL)** **VLDL-c (mg/dL)**	44.87 ± 1.90 14.8 ± 0.51	42.91 ± 2.20 14.5 ± 0.72	59.83 ± 1.98^a^ 23.76 ± 0.69^a^	48.51 ± 2.01^a,b^ 18.2 ± 0.66^a,b^

### 3.7 Impact of Hg and SDSCS treatment on oxidative stress biomarkers

#### 3.7.1 Lipid peroxidation

Hg exposure significantly increased lipid peroxidation in both liver and kidney tissues, as evidenced by elevated malondialdehyde (MDA) levels compared with the control group (p < 0.05; Table 7A). Co-administration of SDSCS significantly reduced tissue MDA concentrations in both organs compared with the Hg-treated group (p < 0.05), indicating attenuation of Hg-induced oxidative damage. Although MDA levels were markedly decreased following SDSCS treatment, they remained significantly different from those of the control group. No significant differences were observed between the SDSCS-only and control groups (p > 0.05).

**Table 7:** Effect of HgCl₂ and SDSCS on oxidative stress biomarkers in liver and kidney tissues of male Swiss albino mice. (A) Lipid peroxidation (MDA) (B) Total antioxidant capacity (FRAP) Values are expressed as mean ± SEM (n = 10). HgCl₂ (0.5 mg/kg/day) and SDSCS (20 g/kg/day) were administered by oral gavage for 8 weeks. ^a^ p < 0.05 versus Control group, ^b^ p < 0.05 versus Hg group.

A.				
**MDA** (nmol/mg tissue)	**Control**	**SDSCS**	**Hg**	**SDSCS + Hg**
**Liver**	8.52 ± 0.12	8.01 ± 0.11	19.23 ± 0.81^a^	11.5 ± 0.32^a,b^
**Kidney**	10.22 ± 0.34	9.81 ± 0.82	26.31 ± 0.92^a^	13.7 ± 0.81^a,b^

B.				
**FRAP** (μmolFe^2+^/g tissue)	**Control**	**SDSCS**	**Hg**	**SDSCS + Hg**
**Liver**	1.44 ± 0.16	1.46 ± 0.15	0.66 ± 0.15^a^	1.03 ± 0.51^a,b^
**Kidney**	1.78 ± 0.14	1.65 ± 0.12	0.80 ± 0.22^a^	1.13 ± 0.68^a,b^

#### 3.7.2 3.6.2. Total antioxidant capacity

Hg exposure significantly decreased total antioxidant capacity in both liver and kidney tissues, as determined by the ferric reducing antioxidant power (FRAP) assay (p < 0.05; Table 7B**).** Co-treatment with SDSCS significantly increased FRAP values compared with the Hg-treated group (p < 0.05), demonstrating partial restoration of antioxidant capacity. However, antioxidant levels remained lower than those observed in the control group. The SDSCS-only group showed no significant differences in FRAP values compared with the control group (p > 0.05). Collectively, these findings indicate that SDSCS markedly alleviated Hg-induced oxidative stress by reducing lipid peroxidation and partially restoring tissue antioxidant capacity (Table 7).

### 3.8 impact of Hg and SDSCS treatment on histoarchitecture of liver and kidney

**Fig. 3:**
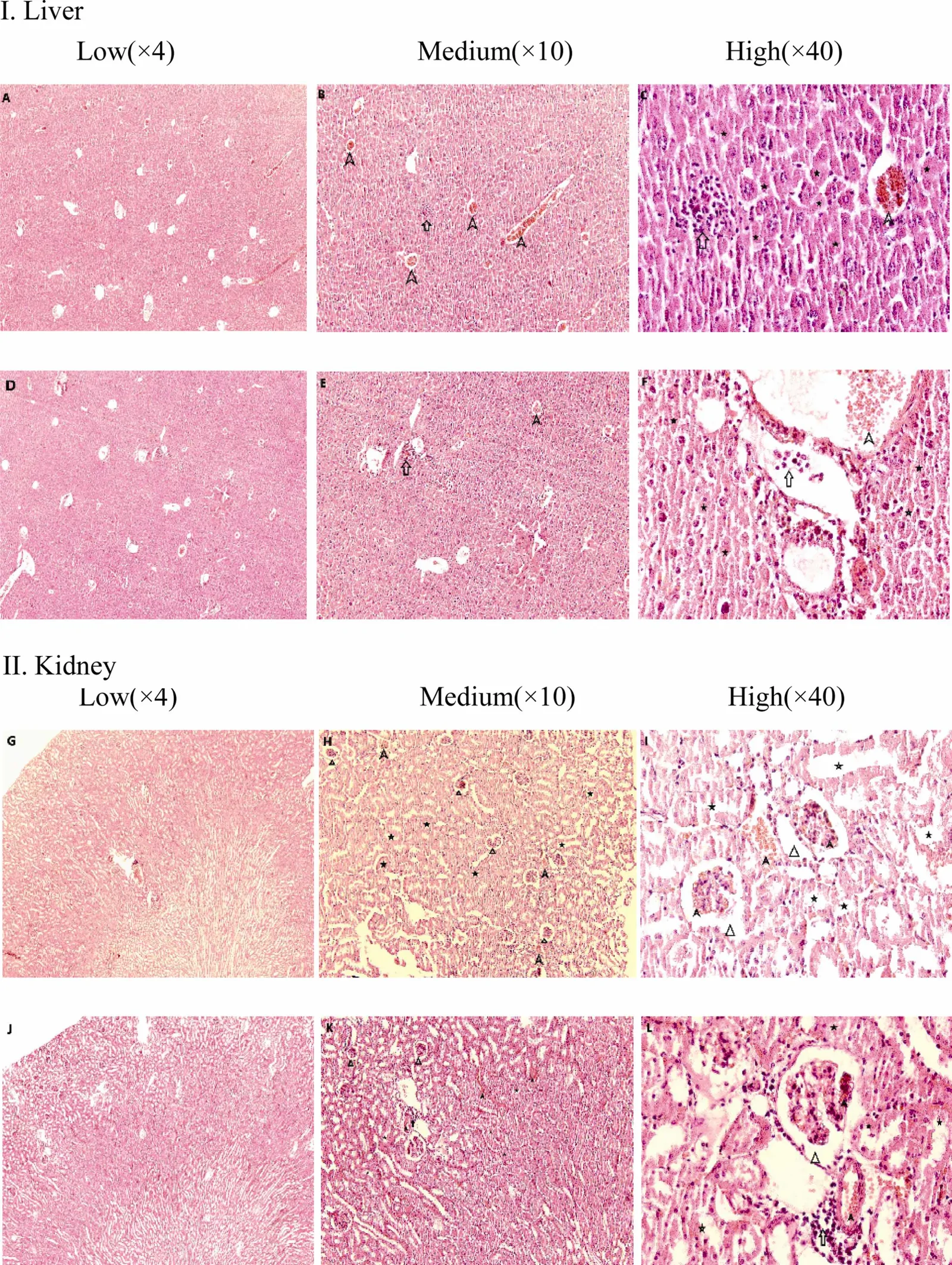
Representative histopathological changes in liver and kidney tissues following Hg exposure and SDSCS treatment (H&E staining). Liver sections (A–F) and kidney sections (G–L) are shown at low (4 ×), medium (10 ×), and high (40 ×) magnifications. Hg exposure induced hepatocellular degeneration and necrosis ( ★), sinusoidal congestion (▲), inflammatory cell infiltration (↑), tubular epithelial degeneration and necrosis (★), and glomerular degeneration (△). Co-treatment with SDSCS markedly reduced the severity of these histopathological alterations and preserved tissue architecture.

**Fig. 4:**
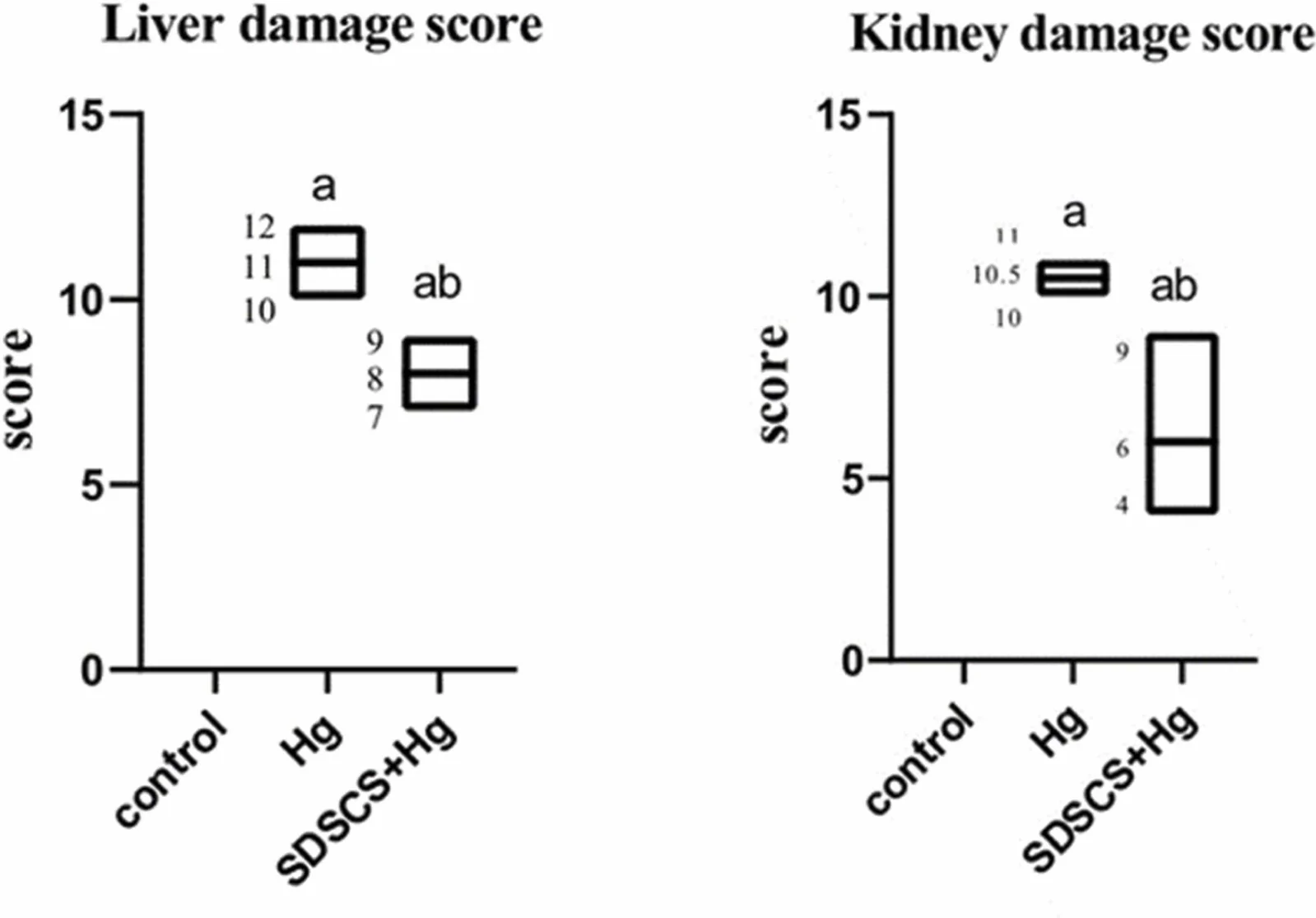
Histopathological injury scores of liver and kidney tissues. Data are presented as median (minimum–maximum)**.** Statistical comparisons were performed using the Kruskal–Wallis test followed by the Mann–Whitney U post hoc test. ^a^ p < 0.05 versus Control group; ^*b*^ p < 0.05 versus Hg group.

Histopathological examination revealed normal hepatic and renal architecture in both the control and SDSCS-only groups, with no detectable pathological alterations (Fig. 3). In contrast, Hg exposure produced marked histopathological lesions in both organs. Liver sections from Hg-treated mice exhibited sinusoidal and central vein congestion, hepatocellular degeneration and necrosis, inflammatory cell infiltration within the portal areas, and bile duct hyperplasia (Fig. 3A–C). Kidney sections showed glomerular degeneration, tubular epithelial degeneration and necrosis, tubular desquamation, vascular congestion, and inflammatory cell infiltration (Fig. 3G–I). Co-administration of SDSCS markedly attenuated these histopathological alterations. Liver sections demonstrated improved hepatic architecture with reduced hepatocellular degeneration, necrosis, and inflammatory infiltration (Fig. 3D–F), whereas kidney sections exhibited reduced tubular degeneration, preservation of glomerular structure, and less inflammatory infiltration compared with the Hg-treated group (Fig. 3J–L). Histopathological scoring confirmed these microscopic observations. Hg exposure significantly increased liver and kidney injury scores compared with the control group (p < 0.05; Fig. 4), whereas SDSCS co-treatment significantly reduced tissue damage scores compared with the Hg-treated group (p < 0.05). No significant histopathological alterations were observed in the SDSCS-only group.

### 3.9 Mercury concentrations in liver and kidney tissues

Subchronic Hg exposure resulted in significant mercury accumulation in both liver and kidney tissues compared with the control group (p < 0.05; Table 8). Mercury concentrations were higher in the kidney than in the liver, indicating greater tissue accumulation in the renal tissue following Hg exposure.

**Table 8:** Mercury concentrations in liver and kidney tissues of male Swiss albino mice following HgCl₂ and SDSCS treatment. Mercury concentrations were determined by hydride-generation atomic absorption spectrophotometry (HG-AAS) and are expressed as ppb dry tissue weight. Values are presented as mean ± SEM (n = 10). ND: Not detected (below the limit of detection) *p < 0.05 versus Hg group.

Tissue	Control		SDSCS	Hg	Hg + SDSCS
Liver	ND		ND	1179 ± 95	792 ± 68*
Kidney	ND		ND	2757 ± 210	1820 ± 125*

Co-administration of SDSCS significantly reduced mercury concentrations in both organs compared with the Hg-treated group (p < 0.05). The reduction in tissue mercury burden was more pronounced in the kidney than in the liver. Mercury was not detected (ND) in the liver or kidney tissues of the control and SDSCS-only groups. Overall, these findings demonstrate that SDSCS significantly decreased Hg accumulation in both hepatic and renal tissues following subchronic exposure (Table 8).

### 3.10 Integrated Z-score and correlation analyses

**Fig. 5:**
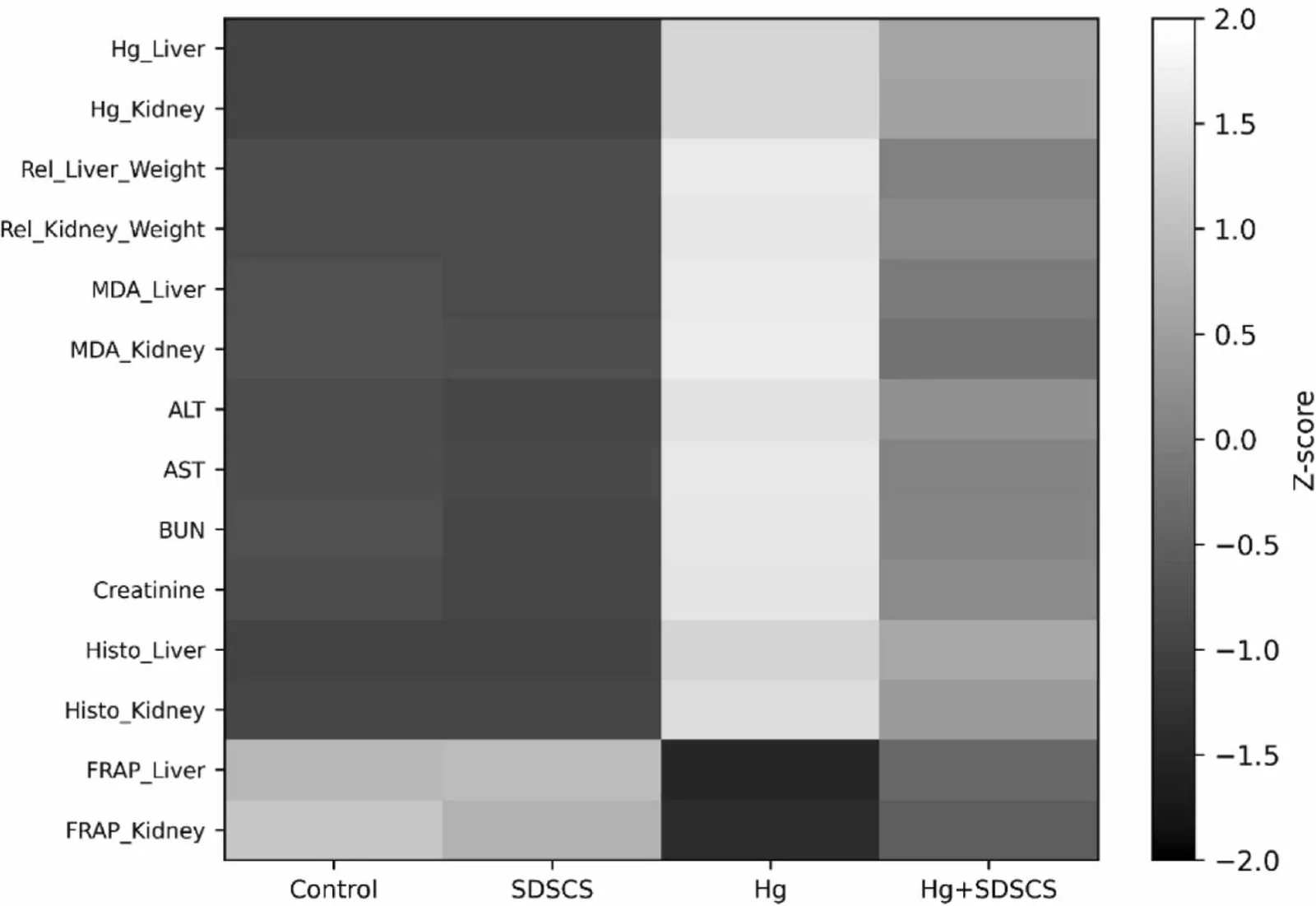
Integrated Z-score heatmap of experimental parameters following Hg exposure and SDSCS treatment. The heatmap illustrates standardized (Z-score) values of tissue mercury concentrations, relative organ weights, oxidative stress biomarkers, biochemical indices, antioxidant capacity, and histopathological injury scores across experimental groups. Higher and lower standardized values are represented according to the grayscale intensity.

The integrated Z-score heatmap demonstrated clear separation of the experimental groups based on the measured toxicological endpoints (Fig. 5). The Hg-treated group exhibited the highest standardized values for tissue mercury concentrations, relative liver and kidney weights, lipid peroxidation (MDA), liver and kidney function biomarkers, and histopathological injury scores, whereas FRAP values showed the lowest standardized values. Co-administration of SDSCS shifted the overall multivariate profile toward that of the control group, with lower standardized values for mercury burden, oxidative stress markers, biochemical indices, relative organ weights, and histopathological scores, together with higher FRAP values than those observed in the Hg-treated group.

**Fig. 6:**
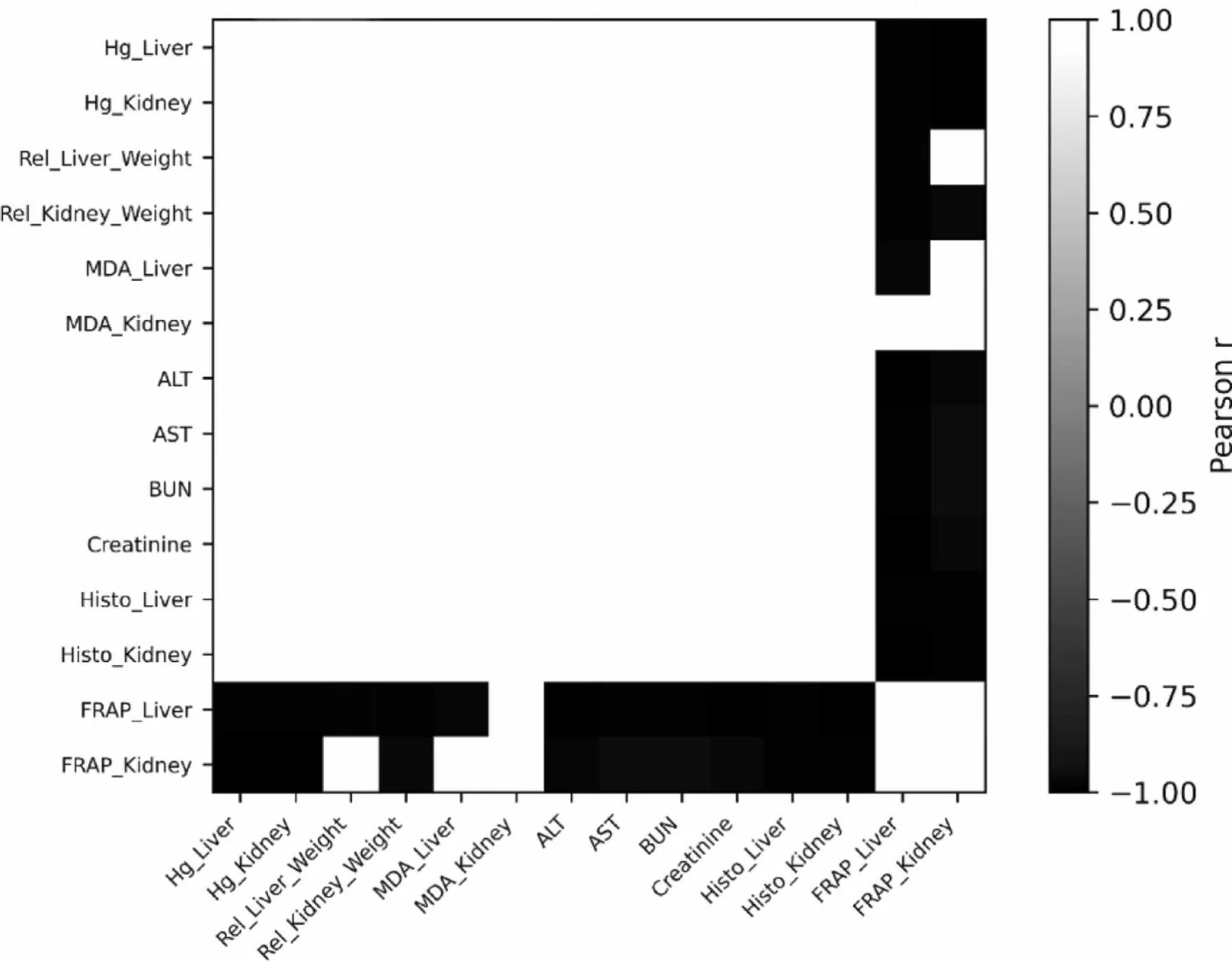
Pearson correlation heatmap of measured hepatorenal toxicity parameters. Positive and negative Pearson correlation coefficients among tissue mercury concentrations, relative organ weights, oxidative stress biomarkers, biochemical parameters, antioxidant capacity, and histopathological injury scores are presented. Only statistically significant correlations **(**p < 0.05**)** are displayed.

## 4 Discussion

Mercury is a persistent environmental pollutant with a well-established capacity to induce oxidative damage, disrupt cellular metabolism, and cause progressive hepatorenal injury [30], [39], [56]. The present study provides comprehensive evidence that sodium dodecyl sulfate–modified chitosan (SDSCS) markedly attenuates subchronic mercury-induced toxicity in mice. By integrating in vitro mercury-binding experiments, tissue mercury quantification, biochemical and hematological assessments, histopathological evaluation, and multivariate statistical analyses, this study offers strong experimental evidence supporting potential mechanisms through which SDSCS mitigates mercury toxicity.

The mercury dose employed in the present study (HgCl₂, 0.5 mg/kg/day for 8 weeks) was selected to model subchronic cumulative exposure rather than acute intoxication. Although this dose exceeds typical daily human exposure when compared on a simple mg/kg basis, rodents possess a higher metabolic rate, more rapid renal clearance, and a shorter biological half-life for inorganic mercury than humans. Consequently, higher per-kilogram doses are generally required in rodents to achieve tissue mercury burdens and pathological outcomes comparable to those observed during chronic occupational or environmental exposure in humans. When adjusted using body surface area–based allometric scaling, the administered dose corresponds to a substantially lower human equivalent dose (approximately 0.04 mg/kg/day), which overlaps with cumulative exposure ranges reported in chronically exposed populations. Moreover, the prolonged dosing regimen was specifically designed to mimic repeated low-level exposure associated with progressive tissue accumulation, thereby increasing the translational relevance of this experimental model while acknowledging that direct extrapolation from rodents to humans requires further pharmacokinetic and toxicological investigation [5], [16], [40].

The in vitro mercury-binding assay demonstrated that SDSCS effectively sequestered mercury ions in a concentration-dependent manner. This behavior is likely attributable to the abundance of free amino and hydroxyl functional groups within the modified chitosan backbone, which are capable of coordinating with mercury ions through multiple binding interactions. The tendency toward saturation at higher SDSCS concentrations suggests that binding efficiency eventually becomes limited by mercury availability rather than polymer concentration. Although equilibrium kinetics were not specifically investigated, these findings provide direct experimental evidence supporting the observed reduction in tissue mercury accumulation following SDSCS administration. The ability of functionalized nanocomposite materials to efficiently scavenge Hg(II) from aqueous systems has likewise been demonstrated, supporting the broader feasibility of engineered functional materials for mercury sequestration [46], [53]. Collectively, the in vitro data are consistent with the hypothesis that direct mercury binding contributes to the protective effects of SDSCS while complementing its antioxidant properties [14], [18], [49]. Previous investigations have demonstrated that chemically modified chitosan derivatives exhibit superior adsorption capacity for heavy metals compared with native chitosan because surface modification increases the availability of active binding sites, improves dispersion characteristics, and enhances interactions between polymer functional groups and metal ions. Studies of modified chitosan derivatives have demonstrated their capacity to adsorb divalent heavy-metal ions such as nickel, zinc, and cadmium, supporting the broader utility of functionalized chitosan matrices as heavy-metal-binding materials [54]. Consistent with this concept, crosslinked magnetic chitosan has been successfully investigated for the removal of trace heavy metals from aqueous solutions, further demonstrating the capacity of structurally modified chitosan materials to function as effective metal-binding matrices [48]. Surfactant modification with sodium dodecyl sulfate may further improve these physicochemical properties by increasing accessibility of amino groups and facilitating metal complex formation, thereby enhancing adsorption efficiency. Similar improvements have previously been reported for the removal of various heavy metals using SDS-modified chitosan-based materials [10], [17].

Consistent with these mechanistic observations, the present study demonstrates that SDSCS significantly attenuated subchronic mercury-induced toxicity in mice, particularly with respect to hepatorenal function, oxidative stress parameters, tissue mercury accumulation, and systemic physiological status [13], [24], [25]. Mercury intoxication is frequently associated with impaired body weight gain together with enlargement of detoxification organs due to progressive cellular injury and inflammation. In agreement with previous experimental studies, mercury-treated animals exhibited marked body weight loss accompanied by increased liver and kidney indices [20], [42], [43]. The reduction in body weight is likely attributable to systemic toxicity resulting from decreased food consumption, gastrointestinal dysfunction, impaired nutrient utilization, mitochondrial dysfunction, oxidative stress, and inhibition of multiple metabolic enzymes, ultimately leading to negative energy balance and growth retardation [36], [57], [58]. Comparable alterations in body weight and lipid peroxidation have also been reported in cadmium-exposed rats, in which anthocyanin treatment attenuated heavy-metal-associated metabolic and oxidative disturbances [36]. Following SDSCS administration, body weight gain was significantly improved toward control values, suggesting an overall reduction in systemic toxic burden. Although the present study did not directly evaluate gastrointestinal mercury absorption, the concurrent reduction in tissue mercury accumulation supports the possibility that SDSCS may decrease mercury bioavailability through gastrointestinal binding, thereby contributing to improved metabolic homeostasis. Nevertheless, this mechanism should be considered a plausible interpretation rather than definitive evidence and warrants direct investigation in future studies [33]. The significant increases in absolute and relative liver and kidney weights observed in mercury-exposed animals represent characteristic pathological responses to chronic heavy metal toxicity [19], [32]. Because the liver and kidneys serve as the primary organs responsible for mercury metabolism, distribution, and excretion, they are particularly susceptible to mercury accumulation and subsequent tissue injury. Previous experimental evidence in mice chronically exposed to inorganic mercury has demonstrated significant renal disturbances involving nitric oxide synthase (NOS) activity and zinc–metallothionein homeostasis, further supporting the particular susceptibility of the kidney to chronic inorganic mercury exposure [41]. The elevated organ indices observed in the mercury group most likely reflect hepatocellular hypertrophy, tissue edema, inflammatory cell infiltration, vascular congestion, and tubular degeneration [1], [12]. SDSCS administration significantly reduced both absolute and relative organ weights toward control values, indicating attenuation of tissue injury and compensatory organ enlargement. These macroscopic improvements closely paralleled the observed reductions in tissue mercury accumulation, oxidative stress biomarkers, and histopathological lesions. Although decreased gastrointestinal mercury sequestration represents one possible explanation for these findings, additional pharmacokinetic studies are required to directly confirm this proposed mechanism

Subchronic mercury exposure markedly enhanced lipid peroxidation while simultaneously impairing antioxidant defenses in hepatic and renal tissues, consistent with previous reports [7], [32], [42]. Mercury-induced oxidative stress arises through multiple mechanisms, including direct reactive oxygen species (ROS) generation, depletion of intracellular glutathione through Hg–GSH complex formation, inhibition of antioxidant enzymes, mitochondrial dysfunction, displacement of essential trace metals, and activation of inflammatory signaling pathways [13], [22], [57], [56]. Evidence from mercury-exposed occupational populations has likewise demonstrated an association between mercury exposure and oxidative-stress alterations, supporting the biological relevance of redox imbalance as an important component of mercury toxicity [60]. The resulting imbalance between ROS production and antioxidant capacity promotes lipid peroxidation, protein oxidation, mitochondrial impairment, DNA damage, and ultimately cell death [15], [22], [57]. Importantly, previous experimental evidence has demonstrated that chitosan can attenuate heavy-metal-induced oxidative stress and renal injury, providing a relevant precedent for the antioxidant and tissue-protective effects observed with SDSCS in the present study [38]. Although the present study evaluated malondialdehyde (MDA) and total antioxidant capacity (TAC) rather than specific antioxidant enzymes or inflammatory mediators, the observed biochemical changes are consistent with previously described mechanisms of mercury-induced oxidative injury [26]. Therefore, the proposed involvement of antioxidant, anti-inflammatory, and mitochondrial protective pathways should be regarded as biologically plausible mechanisms supported by the literature rather than mechanisms directly demonstrated in the present study.

In the liver, mercury-induced oxidative stress was reflected by elevated MDA levels together with significant increases in serum ALT, AST, ALP, and bilirubin, accompanied by reductions in total protein and albumin, indicating impaired hepatocellular integrity and synthetic function [12], [19], [20]. Similarly, renal dysfunction was evidenced by elevated serum creatinine and blood urea nitrogen (BUN), reflecting impaired glomerular filtration and tubular injury [32], [41]. The close association between HgCl₂ exposure, oxidative stress, and hepatic and renal injury reported in previous experimental work is consistent with the coordinated biochemical improvements observed following SDSCS administration in the present study [43]. Administration of SDSCS significantly improved these biochemical parameters, shifting most values toward those observed in the control group rather than completely restoring normal physiological status**.** These findings indicate substantial preservation of hepatic and renal function following SDSCS treatment.

Subchronic mercury exposure also induced significant disturbances in glucose and lipid metabolism, as evidenced by hyperglycemia, hypercholesterolemia, hypertriglyceridemia, reduced HDL-c, and elevated LDL-c and VLDL-c concentrations. These findings are consistent with previous studies demonstrating that mercury disrupts systemic metabolic homeostasis through oxidative stress, mitochondrial dysfunction, and impairment of insulin signaling pathways [11], [21], [57]. Evidence from other heavy-metal exposure models similarly indicates that metal-induced oxidative stress can be accompanied by disturbances in lipid metabolism and lipid peroxidation, supporting the broader association between heavy-metal toxicity and metabolic dysregulation [36]. Oxidative damage induced by mercury may reduce pancreatic β-cell function, interfere with glucose uptake in peripheral tissues, and impair hepatic glucose metabolism, thereby contributing to the development of hyperglycemia. In parallel, mercury-induced oxidative stress can alter lipid synthesis, transport, and degradation, leading to dyslipidemia characterized by increased circulating cholesterol and triglycerides together with unfavorable lipoprotein profiles. The observed reduction in HDL-c accompanied by increased LDL-c and VLDL-c further suggests impairment of lipid transport and enhanced susceptibility to lipid peroxidation. Such alterations are considered important contributors to cardiovascular and metabolic complications associated with chronic mercury exposure and have been reported in both experimental animals and exposed human populations [45], [57]. Administration of SDSCS significantly attenuated these metabolic disturbances. Animals receiving SDSCS together with mercury exhibited significantly lower blood glucose, total cholesterol, triglycerides, LDL-c, and VLDL-c concentrations, together with partial restoration of HDL-c levels compared with the mercury-treated group. Importantly, these parameters shifted toward control values rather than returning completely to normal, indicating substantial but incomplete metabolic recovery following SDSCS treatment. The mechanisms underlying these beneficial effects were not directly investigated in the present study. However, the reduction in tissue mercury accumulation together with improvement of oxidative stress indices suggests that decreased systemic mercury burden may contribute to preservation of normal metabolic regulation. In addition, previous studies have demonstrated that chitosan and its derivatives may exert favorable effects on lipid metabolism by reducing intestinal lipid absorption, binding bile acids, and improving lipid homeostasis, while their antioxidant properties may indirectly preserve glucose metabolism [16], [34], [35]. Because these mechanisms were not specifically evaluated in the present study, they should be regarded as biologically plausible explanations supported by previous literature rather than experimentally confirmed mechanisms. Overall, the improvement in glucose and lipid metabolism observed following SDSCS administration extends its protective effects beyond hepatorenal toxicity and supports the concept that limiting mercury bioavailability may contribute to preservation of systemic metabolic homeostasis during chronic mercury exposure.

Histopathological evaluation further corroborated these biochemical observations. Mercury exposure induced extensive hepatocellular degeneration, coagulative necrosis, inflammatory infiltration, and renal tubular degeneration, all of which were accompanied by significantly higher histopathological injury scores. Importantly, the semi-quantitative histopathological scores closely paralleled the biochemical indices of liver and kidney dysfunction, providing complementary structural and functional evidence of mercury-induced organ injury. SDSCS co-treatment markedly preserved tissue architecture and significantly reduced histopathological lesion scores in both organs. These improvements are consistent with reduced mercury accumulation and attenuation of oxidative injury; however, because inflammatory cytokines, apoptotic markers, and mitochondrial function were not directly measured, these mechanisms remain inferential and require confirmation in future mechanistic studies [17], [56].

Beyond organ-specific toxicity, mercury exposure caused pronounced hematological disturbances, including anemia, leukocytosis, and thrombocytopenia, consistent with previous reports [11], [46]. These alterations most likely resulted from oxidative injury to erythrocyte membranes, impaired erythropoiesis, inflammatory activation, and bone marrow dysfunction. SDSCS significantly ameliorated these hematological abnormalities, further supporting its capacity to reduce systemic mercury toxicity. Chitosan oligosaccharide has also demonstrated beneficial effects on hematological alterations associated with heavy-metal exposure, supporting the possibility that chitosan-based materials may contribute to systemic protection against metal-induced hematotoxicity [29]. Although hematological recovery occurred concurrently with reductions in tissue mercury burden and oxidative stress, these findings further support the overall protective profile of SDSCS rather than indicating a specific hematopoietic mechanism.

One of the most important findings of the present study was the direct quantification of mercury accumulation in target organs. Mercury concentrations increased markedly in both liver and kidney tissues following subchronic HgCl₂ exposure, with significantly higher concentrations detected in the kidneys, reflecting their central role in mercury filtration, tubular uptake, and excretion [1], [45]. Co-administration of SDSCS reduced hepatic mercury accumulation by 32.8% and renal mercury accumulation by 34.0%, providing quantitative evidence of reduced tissue mercury burden. These findings are fully consistent with the concentration-dependent mercury-binding activity demonstrated in the in vitro assay and strongly support the hypothesis that SDSCS decreases systemic mercury bioavailability. Recent work demonstrating protection against dietary methylmercury toxicity through an engineered gastrointestinal bacterium further illustrates the potential of gut-directed interventions to limit mercury exposure before systemic distribution [59]. Nevertheless, because mercury absorption, distribution, metabolism, and excretion were not directly investigated, the precise pharmacokinetic mechanisms responsible for the reduced tissue mercury concentrations remain to be elucidated.

Furthermore, the observed protective effects of SDSCS compare favorably with previous studies evaluating other natural antioxidants and biopolymer-based detoxification strategies, including selenium compounds, quercetin, curcumin, thymoquinone, taurine, melatonin and native chitosan derivatives, which similarly reduced oxidative stress and mercury-induced tissue injury primarily through antioxidant and metal-binding mechanisms [9], [21], [28], [31], [34], [35], [37], [47], [52], [61]. In particular, the demonstrated protective effect of chitosan against lead-induced renal oxidative stress provides supporting evidence that chitosan-based biomaterials may possess intrinsic protective properties in heavy-metal exposure models [38], [55]. Compared with conventional chelating agents such as dimercaptosuccinic acid (DMSA), 2,3-dimercapto-1-propanesulfonic acid (DMPS), and British anti-Lewisite (BAL) [23], SDSCS may offer potential advantages as a biocompatible biomaterial capable of simultaneously reducing mercury bioavailability while providing antioxidant support. More broadly, physical, chemical, and biological approaches have been investigated for the removal and remediation of heavy metals from contaminated environments, highlighting the importance of developing effective and sustainable metal-sequestration strategies [51]. However, direct comparative studies are required before any conclusions regarding therapeutic superiority can be drawn.

Finally, integrated Z-score and correlation heatmap analyses provided a comprehensive overview of the complex relationships among mercury accumulation, oxidative stress, biochemical dysfunction, organ morphometry, and histopathological injury. Unlike conventional univariate statistical analyses, these multivariate approaches enabled simultaneous visualization of coordinated biological responses across multiple endpoints, thereby facilitating identification of toxicity-associated patterns that may not be readily apparent when individual variables are analyzed separately [34], [40], [42], [50]. The strong positive correlations observed between tissue mercury concentrations, oxidative stress biomarkers, relative organ weights, biochemical indices of liver and kidney dysfunction, and histopathological injury scores support the concept that mercury-induced toxicity represents an interconnected pathological network rather than isolated alterations in individual biomarkers. Conversely, antioxidant capacity showed consistent negative correlations with mercury accumulation and tissue injury, further emphasizing the central role of oxidative imbalance in mercury-induced hepatorenal toxicity. SDSCS markedly disrupted this toxicity network by reducing tissue mercury burden while improving antioxidant status and organ function, resulting in coordinated improvements across molecular, biochemical, structural, and morphometric endpoints. These findings highlight the added biological value of integrated multivariate analyses for evaluating complex toxicological responses and may facilitate future assessment of biomaterial-based detoxification strategies [27].

Collectively, the present study provides strong experimental evidence supporting the potential protective effects of SDSCS against subchronic mercury-induced hepatorenal toxicity. These protective effects are consistent with reduced mercury bioavailability and improved redox homeostasis; however, the precise molecular mechanisms were not directly investigated and therefore require further confirmation. A major strength and novelty of this study lies in the integration of concentration-dependent in vitro mercury-binding assays with in vivo tissue mercury quantification, biochemical and hematological evaluation, histopathological assessment, and multivariate Z-score and correlation heatmap analyses. Together, these complementary approaches provide strong experimental evidence supporting potential mechanisms through which SDSCS may mitigate mercury-induced toxicity while offering a comprehensive toxicological evaluation beyond conventional single-endpoint analyses.

Several limitations should be acknowledged, including the exclusive use of male mice, evaluation of a single mercury dose and a single SDSCS dose, restriction to inorganic mercury exposure, and the absence of direct molecular analyses of inflammatory, apoptotic, mitochondrial, and antioxidant signaling pathways. These limitations should be addressed in future investigations. Future studies should evaluate the pharmacokinetics, long-term safety, dose-response relationships, efficacy against different mercury species, and therapeutic performance of SDSCS in additional experimental models. Such investigations will be essential for determining its translational potential as a biopolymer-based strategy for reducing heavy metal toxicity.

## CRediT authorship contribution statement

**Sepide Safari:** Writing – original draft, Visualization, Investigation, Data curation. **Amir Moghaddam Jafari:** Writing – review & editing, Supervision, Project administration, Formal analysis, Conceptualization. **Hossein Nourani:** Investigation, Data curation.

## Funding

This work was supported by the veterinary faculty and the Vice-Chancellor of Research, Ferdowsi University of Mashhad, Mashhad, Iran.

## Declaration of Competing Interest

The authors declare that they have no known competing financial interests or personal relationships that could have appeared to influence the work reported in this paper.

## Data Availability

Data will be made available on request.
